# Association between Plant-based Diet and Risk of Chronic Diseases and All-Cause Mortality in Centenarians in China: A Cohort Study

**DOI:** 10.1016/j.cdnut.2023.102065

**Published:** 2023-12-19

**Authors:** Lei Yuan, QinQin Jiang, Yinghong Zhai, Zhe Zhao, Yijun Liu, Fangyuan Hu, Yi Qian, Jinhai Sun

**Affiliations:** 1 Department of Health Management, Faculty of Military Health Service, Naval Medical University, Shanghai, China; 2 Clinical Research Unit, School of Medicine, Shanghai 9th People's Hospital Affiliated to Shanghai JiaoTong University, Shanghai, China; 3 Department of Medical Service, Naval Hospital of Eastern Theater, Zhoushan, China; 4 College of Health Management, Southern Medical University, Guangzhou, China

**Keywords:** plant-based diet, animal-based diet, all-cause mortality, chronic diseases, centenarians, China

## Abstract

**Background:**

Numerous studies have suggested the health benefits of a plant-based dietary pattern. However, whether this dietary pattern is associated with health benefits for centenarians remains unexplored. Our study aimed to investigate the correlation between 16 widely consumed Chinese food items and the incidence rates of chronic diseases and all-cause mortality among centenarians.

**Methods:**

We conducted a dietary survey on 3372 centenarians with an average age of 102.33 y in China. After rigorous screening, we identified 2675 centenarians, who underwent a 10-y follow-up study with all-cause mortality as the primary outcome. We developed 6 dietary patterns on the basis of the food consumption frequency of each participant. To model the impact of missing values, we employed multiple imputation methods, verifying the robustness of models.

**Results:**

The overall plant-based diet index (PDI), healthy plant-based diet index (hPDI), unhealthy plant-based diet index (uPDI), healthy plant-based foods index (HPF), unhealthy plant-based foods index (uHPF), and animal-based foods index (AF) scores among centenarians in China were 46.95 ± 6.29, 44.43 ± 5.76, 51.09 ± 6.26, 21.63 ± 4.79, 9.91 ± 2.41, and 14.59 ± 3.58, respectively. High scores of PDI, hPDI, and HPF were associated with a lower risk of chronic diseases. In the 10-y follow-up study, 92.90% of centenarians have died. The high scores of the PDI (HR_PDI_ = 0.81), hPDI (HR_hPDI_ = 0.79), and HPF (HR_HPF_ = 0.81) scores were significantly associated with a lower risk of death compared with the low scores. Conversely, the high AF score (HR_AF_ = 1.17) was significantly associated with a higher risk of death compared with the low scores.

**Conclusion:**

Despite the fact that a higher score in both a predominantly plant-based dietary pattern and a healthy dietary pattern can decrease the death among centenarians, not all HPFs have this effect. A higher AF predicted a higher risk of mortality, whereas higher PDI, hPDI, and HPF were associated with a lower risk of mortality among Chinese centenarians.

## Introduction

Plant-based dietary patterns are becoming increasingly popular worldwide, and most evidence suggests that this type of dietary pattern can help elderly individuals reduce the risk of chronic diseases, such as stroke, heart disease, and diabetes, as well as all-cause mortality rates [[1], [2], [3], [4], [5]]. Several studies on centenarians indicate that their exceptional longevity results from relatively late onset of major life-threatening ailments (such as stroke, cancer, ischemic heart disease, diabetes, dementia, and osteoporosis) [[6], [7], [8], [9], [10]], and slower aging rates than the general population. However, the existing dietary studies on Chinese centenarians mainly rely on cross-sectional surveys [[11], [12], [13]], with 1 study employing a follow-up investigation to verify the incidence of cognitive impairment [14]. The lack of long-term tracking of the effects of different foods on the lifespan extension of centenarians in this population [15,16], coupled with the relatively small population of centenarians in low-income countries, resulted in a focus on developed countries, such as the United Kingdom, United States, Canada, and Japan for long-term dietary follow-up investigations [[17], [18], [19], [20], [21]]. To the best of our knowledge, there have been no reports on long-term dietary follow-up investigations of Chinese centenarians.

Our objective was to assess the relationship between chronic diseases and all-cause mortality and different dietary patterns, using survey data collected from centenarians in China [22]. Following the common classification methods used in current research, we have categorized 16 types of food intake frequencies into 3 composite dietary patterns [23,24]: plant-based dietary index (PDI), healthy plant-based dietary index (hPDI), and unhealthy plant-based dietary index (uPDI). In addition, we have also calculated the intake scores of the 3 food categories, namely the healthy plant-based foods index (HPF), the unhealthy plant-based foods index (uHPF), and the animal-based foods index (AF), on the basis of the frequency at which different categories of plants are consumed. In our cross-sectional analysis, we primarily analyze the characteristics of the dietary patterns of centenarians in China and their relationship with chronic diseases, whereas in our longitudinal analysis, we mainly observe the all-cause mortality risk associated with different dietary patterns and food categories.

## Methods

### Data resource and exclusion criteria

Our data are derived from the Chinese Longitudinal Healthy Longevity Survey (CLHLS), which is the longest-running longitudinal health survey conducted in China. The project was launched in 1998 and represents 1 of the earliest and largest health tracking projects in China [25]. The study has carried out 8 rounds of surveys in 23 provinces in China, while employing statistical control over the sample size of the oldest-old population by oversampling those aged ≥80, who constitute 67.4% of the total sample. Moreover, the study strives to interview centenarians who voluntarily participate in the research in all of the selected county-level cities. As a result, this study is widely recognized as the world’s largest sample of the elderly population, specifically those aged ≥80 y. This study has received ethical approval from the Institutional Review Board of Peking University (no. IRB00001052-13074); all participants had submitted the written informed consent.

The eligibility criteria for participant inclusion in this study are as follows.

For the cross-sectional analysis: *1*) age ≥100 y, and *2*) complete response to all dietary questions.

For the longitudinal analysis: *1*) meeting the inclusion criteria for the cross-sectional survey, and *2*) possessing at least 1 complete follow-up record.

**FIGURE 1:**
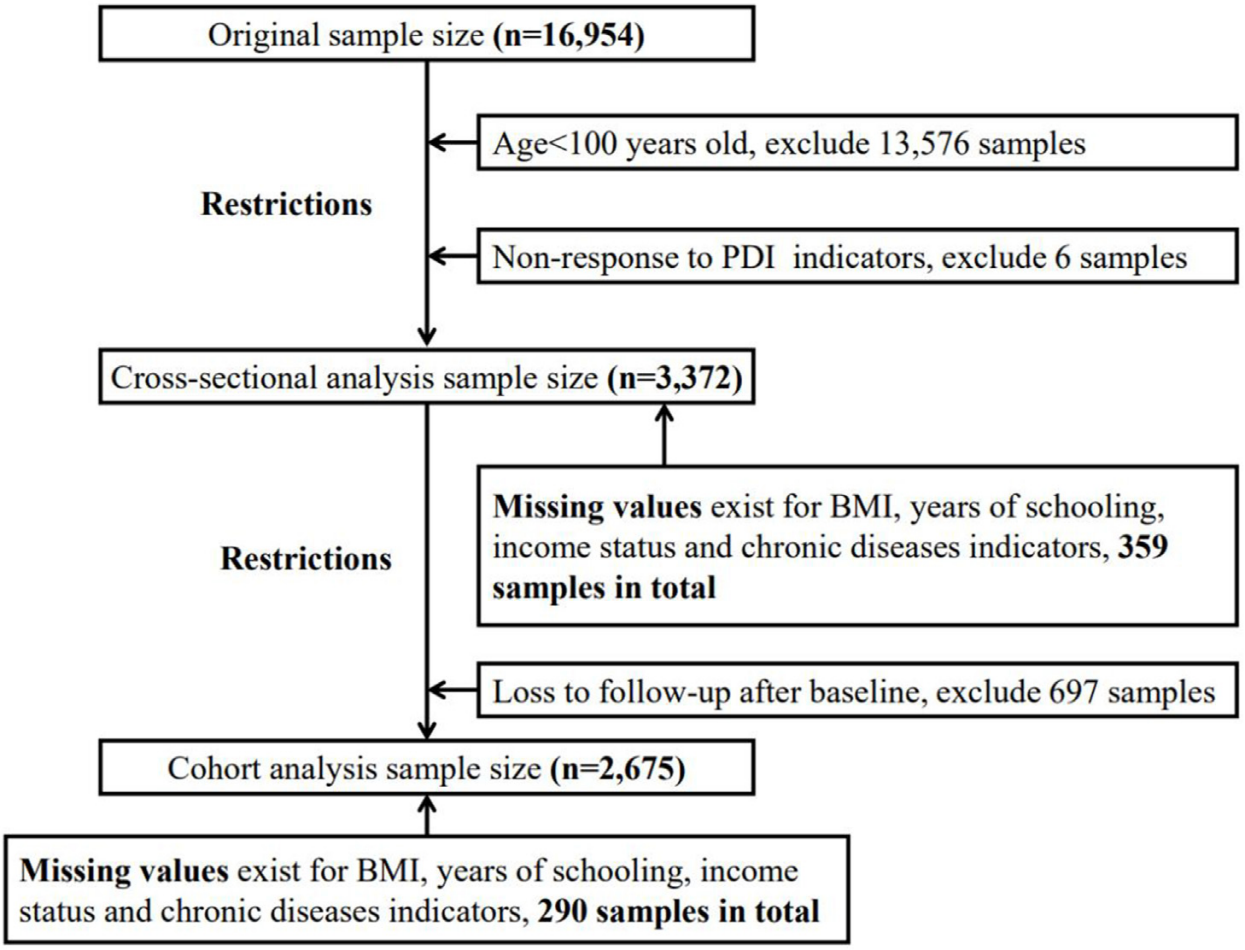
Flowchart of participants in this study.

Data screening is presented in Figure 1. Ultimately, we included 3372 centenarians for cross-sectional analysis and 2675 centenarians for longitudinal analysis.

### Dietary patterns and scores

Taking into consideration the dietary patterns and characteristics of Chinese older adults, as well as referring to dietary scoring methods proposed by some scholars, we selected a total of 16 types of food as indicators for dietary assessment [[22], [23], [24]]. These indicators were classified into 3 categories: HPF, which included 8 types of food such as whole grains, vegetable oil, fresh fruits, fresh vegetables, legumes, garlic, nuts, and tea; unhealthy plant-based foods, which consisted of 3 types of food namely refined grains, preserved vegetables and sugar; and animal-based foods, which included 5 types of food namely meat, fish and aquatic products (fish), milk or dairy products, eggs, and animal fat.

In the CLHLS survey, the frequency of consumption for each type of food was required to be reported, and the validity of this evaluation method has been verified [22,23]. According to the commonly used plant-based dietary classification model among international scholars, we constructed 3 comprehensive dietary patterns and 3 single dietary patterns on the basis of the types of food that Chinese people typically consume. The 3 comprehensive dietary patterns include overall PDI, healthy plant-based diet index (hPDI), and unhealthy plant-based diet index (uPDI). The 3 single dietary patterns include HPF, uHPF, and AF. For the purpose of facilitating the comparison of the HPF, uHPF, and AF dietary patterns on a unified level, we processed the scores of the 3 patterns and named them as HPFs, uHPFs, and AFs (details can be found in Supplementary Method section 1). Different dietary patterns are assigned different scores on the basis of the frequency of consumption of different foods. The frequency of consumption and the scores assigned to each type of food in different dietary patterns are described in detail in Supplemental Table 1.

## Outcomes

In the cross-sectional analysis, the main outcome variables were 9 types of chronic diseases and the number of chronic diseases. The 9 types of chronic diseases included hypertension, diabetes, heart disease, cardiovascular diseases or stroke, chronic respiratory diseases, chronic gastritis, arthritis, dementia, and cholecystitis or cholelithiasis. The number of chronic diseases was categorized into 0, 1, and ≥2 on the basis of the respondents’ self-reported information.

In the longitudinal analysis, the primary outcome variable was mortality. If a participant passed away during the follow-up period, detailed information on the date of death and their major health condition before death was recorded. The information on the date of death was obtained from official death certificates. In cases where no official death certificates were available, information was obtained from the participants’ relatives or neighborhood committees. The validity of mortality information in the CLHLS has been shown to be reliable in other studies [26].

### Covariables

The included covariates were divided into 4 categories [27]: demographic characteristics, socioeconomic characteristics, personal lifestyle, and health status. The demographic characteristics included 5 indicators, namely age, gender, residence, ethnic group, and BMI. The socioeconomic characteristics included 3 indicators: years of schooling, marital status, and income status. Personal lifestyle factors included 3 indicators: current smoker, current alcohol drinker, and regular exercise; health status was based on the 9 types of chronic diseases. The definitions and classifications of each variable can be found in Supplemental Methods section 2.

## Statistics methods

We categorized the PDI, hPDI, uPDI, HPF, uHPF, and AF as ordinal variables using quintiles [22]. In the cross-sectional analysis, the quintiles were calculated on the basis of the data derived from the 3372 centenarians to determine the corresponding quintile for each participant. In the longitudinal analysis, the quintiles were recalculated for the 2675 participants and reclassified accordingly.

The study employed count or mean values to represent the general data. For the cross-sectional analysis, a multiple linear regression model was primarily adopted to analyze the relationship between different dietary patterns scores and chronic diseases; whereas for the longitudinal study, a time-varying Cox proportional hazard model was applied to investigate the association between different dietary patterns quintiles and the overall risk of mortality. Multiple adjustment models with diverse covariates were developed in both cross-sectional and longitudinal studies to verify the link between the 2 factors.

Regarding the missing values of covariates, multiple imputation was employed to simulate the missing data in each category of covariates, as detailed in some previous study [28,29]. Specifically, the missing values of each covariate were filled in using reference values, including age, gender, residence, ethnic group, marital status, current smoker, current alcohol drinker, regular exercise, PDI, hPDI, uPDI, HPF, uHPF, and AF. Each missing value was imputed 10 times, and sensitivity analyses were conducted using both the complete-case analysis and the models with imputed missing values to ensure the robustness of our conclusions.

## Results

### Participate characteristics

The cross-sectional study included a total of 3372 centenarians (Table 1), of which the vast majority were female (80.25%; 2706/3372). The mean age of participants was 102.33 y, with 62.31% residing in rural areas and 93.48% identifying as Han Chinese. In addition, 44.93% had a normal BMI, 85.77% had not received formal education, 96.03% were widowed, and 69.25% perceived their economic status to be average. The longitudinal study consisted of 2675 centenarian participants (Supplemental Table 2), with an average age at follow-up of 102.33 y. The average follow-up period from 2008 to 2018 was 2.47 (2.07) y. Of the total participants, 92.90% (2485/2675) were recorded as deceased.

**TABLE 1:** Information of participants of cross-sectional analysis by PDI quintiles

Variables	Overall	PDI Q1	PDI Q2	PDI Q3	PDI Q4	PDI Q5
Age (y), mean (s.d.)	102.33 (0.04)	102.52 (0.09)	102.37 (0.08)	102.26 (0.09)	102.27 (0.10)	102.19 (0.09)
Gender						
Male	666 (19.75)	126 (16.32)	158 (20.15)	133 (20.18)	116 (20.39)	133 (22.62)
Female	2706 (80.25)	646 (83.68)	626 (79.85)	526 (79.82)	453 (79.61)	455 (77.38)
Residence						
Town	521 (15.45)	112 (14.51)	110 (14.03)	96 (14.57)	94 (16.52)	109 (18.54)
Rural	2101 (62.31)	531 (68.78)	473 (60.33)	408 (61.91)	336 (59.05)	353 (60.03)
City	750 (22.24)	129 (16.71)	201 (25.64)	155 (23.52)	139 (24.43)	126 (21.43)
Ethnic group						
Han	3152 (93.48)	690 (89.38)	742 (94.64)	628 (95.30)	543 (95.43)	549 (93.37)
Other	220 (6.52)	82 (10.62)	42 (5.36)	31 (4.70)	26 (4.57)	39 (6.63)
BMI (kg/m^2^)						
18.5–23.9	1515 (44.93)	305 (39.51)	358 (45.66)	301 (45.68)	272 (47.80)	279 (47.45)
<18.5	1436 (42.59)	371 (48.06)	330 (42.09)	261 (39.61)	228 (40.07)	246 (41.84)
24.0–27.9	167 (4.95)	35 (4.53)	36 (4.59)	43 (6.53)	28 (4.92)	25 (4.25)
≥28.0	49 (1.45)	6 (0.78)	8 (1.02)	9 (1.37)	9 (1.58)	17 (2.89)
Missing	205 (6.08)	55 (7.12)	52 (6.63)	45 (6.83)	32 (5.62)	21 (3.57)
Years of schooling						
0	2892 (85.77)	672 (87.05)	667 (85.08)	570 (86.49)	487 (85.59)	496 (84.35)
1–6	371 (11.00)	80 (10.36)	87 (11.10)	68 (10.32)	60 (10.54)	76 (12.93)
≥7	93 (2.76)	17 (2.20)	24 (3.06)	18 (2.73)	20 (3.51)	14 (2.38)
Missing	16 (0.47)	3 (0.39)	6 (0.77)	3 (0.46)	2 (0.35)	2 (0.34)
Marital status						
Married and living spouse	102 (3.02)	15 (1.94)	24 (3.06)	18 (2.73)	25 (4.39)	20 (3.40)
Windowed	3238 (96.03)	756 (97.93)	756 (96.43)	633 (96.05)	530 (93.15)	563 (95.75)
Other	32 (0.95)	1 (0.13)	4 (0.51)	8 (1.21)	14 (2.46)	5 (0.85)
Income status						
General	2335 (69.25)	528 (68.39)	544 (69.39)	476 (72.23)	385 (67.66)	402 (68.37)
Rich	416 (12.34)	82 (10.62)	91 (11.61)	78 (11.84)	80 (14.06)	85 (14.46)
Poor	605 (17.94)	158 (20.47)	144 (18.37)	101 (15.33)	102 (17.93)	100 (17.01)
Missing	16 (0.47)	4 (0.52)	5 (0.64)	4 (0.61)	2 (0.35)	1 (0.17)
Current Smoker						
No	3138 (93.06)	725 (93.91)	735 (93.75)	618 (93.78)	516 (90.69)	544 (92.52)
Yes	234 (6.94)	47 (6.09)	49 (6.25)	41 (6.22)	53 (9.31)	44 (7.48)
Current alcohol drinker						
No	2972 (88.14)	684 (88.60)	694 (88.52)	586 (88.92)	511 (89.81)	497 (84.52)
Yes	400 (11.86)	88 (11.40)	90 (11.48)	73 (11.08)	58 (10.19)	91 (15.48)
Regular exercise						
No	2852 (84.58)	661 (85.62)	681 (86.86)	567 (86.04)	462 (81.20)	481 (81.80)
Yes	520 (15.42)	111 (14.38)	103 (13.14)	92 (13.96)	107 (18.80)	107 (18.20)
Hypertension						
No	2912 (86.36)	671 (86.92)	675 (86.10)	577 (87.56)	480 (84.36)	509 (86.56)
Yes	384 (11.39)	74 (9.59)	94 (11.99)	75 (11.38)	76 (13.36)	65 (11.05)
Missing	76 (2.25)	27 (3.50)	15 (1.91)	7 (1.06)	13 (2.28)	14 (2.38)
Diabetes						
No	3279 (97.24)	733 (94.95)	771 (98.34)	649 (98.48)	553 (97.19)	573 (97.45)
Yes	25 (0.74)	8 (1.04)	5 (0.64)	3 (0.46)	6 (1.05)	3 (0.51)
Missing	68 (2.02)	31 (4.02)	8 (1.02)	7 (1.06)	10 (1.76)	12 (2.04)
Heart disease						
No	3075 (91.19)	708 (91.71)	719 (91.71)	600 (91.05)	520 (91.39)	528 (89.80)
Yes	234 (6.94)	40 (5.18)	57 (7.27)	53 (8.04)	39 (6.85)	45 (7.65)
Missing	63 (1.87)	24 (3.11)	8 (1.02)	6 (0.91)	10 (1.76)	15 (2.55)
Stroke or CVD						
No	3171 (94.04)	720 (93.26)	728 (92.86)	623 (94.54)	541 (95.08)	559 (95.07)
Yes	145 (4.30)	32 (4.15)	48 (6.12)	31 (4.70)	20 (3.51)	14 (2.38)
Missing	56 (1.66)	20 (2.59)	8 (1.02)	5 (0.76)	8 (1.41)	15 (2.55)
Respiratory disease						
No	3000 (88.97)	678 (87.82)	696 (88.78)	581 (88.16)	514 (90.33)	531 (90.31)
Yes	325 (9.64)	77 (9.97)	81 (10.33)	74 (11.23)	46 (8.08)	47 (7.99)
Missing	47 (1.39)	17 (2.20)	7 (0.89)	4 (0.61)	9 (1.58)	10 (1.70)
Gastric or duodenal ulcer						
No	3156 (93.59)	716 (92.75)	731 (93.24)	623 (94.54)	535 (94.02)	551 (93.71)
Yes	152 (4.51)	32 (4.15)	41 (5.23)	32 (4.86)	23 (4.04)	24 (4.08)
Missing	64 (1.90)	24 (3.11)	12 (1.53)	4 (0.61)	11 (1.93)	13 (2.21)
Arthritis						
No	2671 (79.21)	579 (75.00)	612 (78.06)	541 (82.09)	465 (81.72)	474 (80.61)
Yes	656 (19.45)	182 (23.58)	163 (20.79)	113 (17.15)	95 (16.70)	103 (17.52)
Missing	45 (1.33)	11 (1.42)	9 (1.15)	5 (0.76)	9 (1.58)	11 (1.87)
Dementia						
No	3153 (93.51)	706 (91.45)	732 (93.37)	621 (94.23)	542 (95.25)	552 (93.88)
Yes	170 (5.04)	46 (5.96)	44 (5.61)	36 (5.46)	20 (3.51)	24 (4.08)
Missing	49 (1.45)	20 (2.59)	8 (1.02)	2 (0.30)	7 (1.23)	12 (2.04)
Cholecystitis or cholelith disease						
No	3259 (96.65)	740 (95.85)	761 (97.07)	642 (97.42)	549 (96.49)	567 (96.43)
Yes	52 (1.54)	9 (1.17)	10 (1.28)	12 (1.82)	12 (2.11)	9 (1.53)
Missing	61 (1.81)	23 (2.98)	13 (1.66)	5 (0.76)	8 (1.41)	12 (2.04)
Number of chronic diseases						
0	1880 (55.75)	414 (53.63)	422 (53.83)	372 (56.45)	328 (57.64)	344 (58.50)
1	906 (26.87)	209 (27.07)	211 (26.91)	179 (27.16)	148 (26.01)	159 (27.04)
≥2	452 (13.40)	100 (12.95)	120 (15.31)	93 (14.11)	73 (12.83)	66 (11.22)
Missing	134 (3.97)	49 (6.35)	31 (3.95)	15 (2.28)	20 (3.51)	19 (3.23)

### Scores for different dietary patterns

**FIGURE 2:**
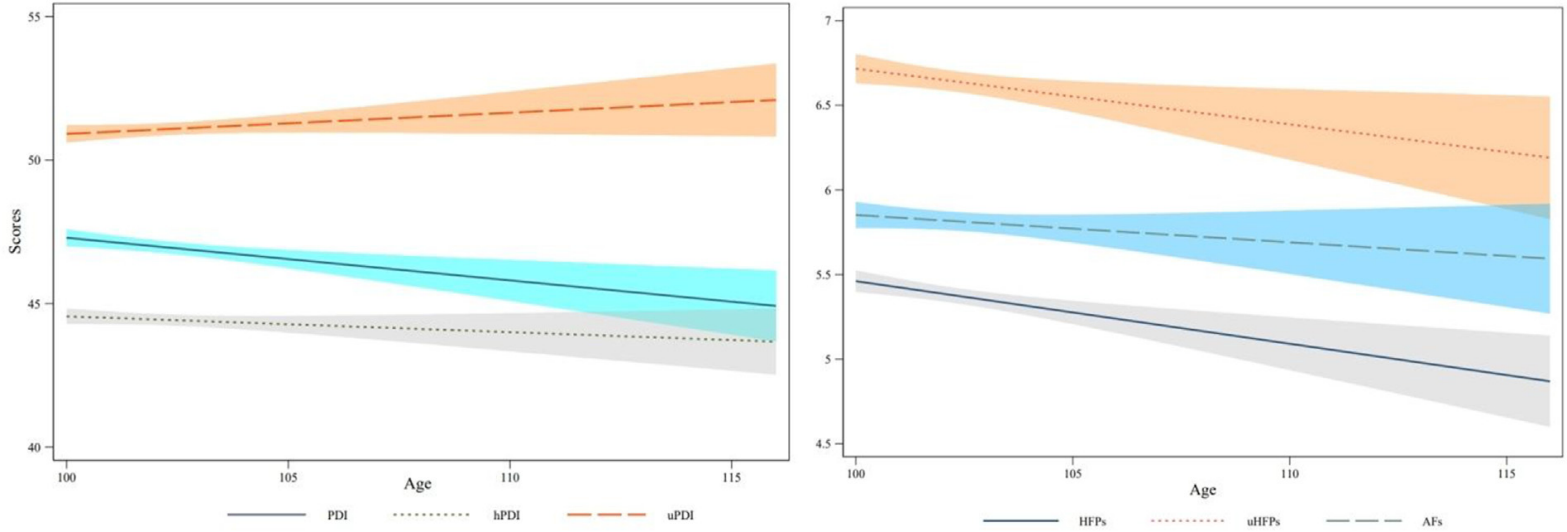
Distribution of dietary scores of Chinese centenarians with age in a cross-sectional survey.

Among the centenarians in China, the scores for PDI, hPDI, uPDI, HPF, uHPF, and AF were 46.95 ± 6.29, 44.43 ± 5.76, 51.09 ± 6.26, 21.63 ± 4.79, 9.91 ± 2.41, and 14.59 ± 3.58, respectively, as presented in Supplemental Table 3. Figure 2 shows the line chart of different dietary pattern scores across age groups. We noted that the uPDI score was the highest among the Chinese centenarians, and it gradually increased with advancing age. Of the 3 single dietary patterns models, the uHPF score was the highest, but the frequency of consumption decreased gradually with age. In summary, the results presented in Figure 2 indicate that the proportion of unhealthy plants in the dietary patterns of Chinese centenarians was relatively high. Supplemental Figures 1 and 2 demonstrate that the strongest associations in cross-sectional and longitudinal studies are between fish and meat, implying that Chinese centenarians who consume pork frequently also have a predilection for fish and meat. Not only that, we also measured the frequency score at which different foods were consumed (Supplemental Table 4); the dietary frequency scores for each food can be found in Supplemental Figures 3–5). Supplemental Table 4 displays the scores and rankings of various food items among centenarian participants in the study. Supplemental Figures 3–5 display the distribution of healthy plant food, unhealthy plant and animal food scores among Chinese centenarians in the survey by age, respectively.

### Association between diet and chronic disease

Table 2 presents the results of a cross-sectional survey investigating the relationship between different dietary patterns and the number of chronic diseases among centenarians, after adjusting for confounding factors through a complete model. The results showed that PDI, hPDI, and HPF dietary patterns were negatively associated with the number of chronic diseases. Specifically, high-PDI scores were found to be negatively correlated with the risk of having multiple chronic diseases (β = −0.76; 95% confidence interval [95% CI]: −1.41, −0.11), high-hPDI scores were negatively correlated with the risk of having one chronic disease (β = −0.80, 95% CI: −1.10, −0.33), and high-HPF scores were negatively correlated with the risk of having more than 1 chronic disease (β = −0.66; 95% CI: −1.16, −0.17). No significant correlations were observed between other dietary patterns and chronic diseases. After adjustment, the results are consistent with previous analysis (Supplemental Table 5).

**TABLE 2:** Results of regression models for the relationship between each dietary pattern score and the number of chronic diseases

Population	β (95% CI)		
	Model 1	Model 2	Model 3
Number of chronic diseases^1^			
0	Ref.	Ref.	Ref.
1	−0.41 (−0.89, 0.07)	−0.40 (−0.88, 0.08)	−0.48 (−0.98, 0.02)
≥2	−0.61 (−1.24, 0.01)	−0.63 (−1.25, −0.01)^6^	−0.76 (−1.41, −0.11) 6
Number of chronic diseases^2^			
0	Ref.	Ref.	Ref.
1	−0.65 (−1.10, −0.21)^7^	−0.64 (−1.08, −0.19)^7^	−0.80 (−1.26, −0.33)^7^
≥2	−0.28 (−0.86, 0.30)	−0.31 (−0.89, 0.27)	−0.49 (−1.10, 0.12)
Number of chronic diseases^3^			
0	Ref.	Ref.	Ref.
1	0.29 (−0.21, 0.78)	0.25 (−0.24, 0.74)	0.30 (−0.19, 0.78)
≥2	0.23 (−0.41, 0.87)	0.29 (−0.35, 0.93)	0.58 (−0.05, 1.21)
Number of chronic diseases^4^			
0	Ref.	Ref.	Ref.
1	−0.36 (−0.74, 0.02)	−0.34 (−0.71, 0.04)	−0.40 (−0.78, −0.02)^6^
≥2	−0.40 (−0.89, 0.09)	−0.44 (−0.93, 0.05)	−0.66 (−1.16, −0.17)^7^
Number of chronic diseases^5^			
0	Ref.	Ref.	Ref.
1	0.12 (−0.07, 0.31)	0.12 (−0.07, 0.31)	0.16 (−0.04, 0.37)
≥2	−0.19 (−0.44, 0.05)	−0.19 (−0.43, 0.06)	−0.16 (−0.41, 0.10)
Number of chronic diseases^6^			
0	Ref.	Ref.	Ref.
1	0.17 (−0.11, 0.45)	0.18 (−0.11, 0.46)	0.24 (−0.05, 0.52)
≥2	0.02 (−0.35, 0.39)	0.002 (−0.36, 0.37)	−0.06 (−0.43, 0.32)

^8^*P* < 0.01.

model 1: unadjusted; model 2: adjusted for age and gender; model 3: adjusted for age, gender, residence, ethnic group, BMI, years of schooling, marital status, income status, current smoker, current alcohol drinker and regular exercise.

Abbreviations: PDI, overall plant-based diet index; hPDI, healthy plant-based diet index; uPDI, unhealthy plant-based diet index; HPF, healthy plant foods index; uHPF, unhealthy foods index; AF, animal foods index; Ref., Reference.

1 The outcome is PDI,

2 The outcome is hPDI

3 The outcome is uPDI

4 The outcome is HPF

5 The outcome is uHPF

6 The outcome is AF

7 *P* < 0.05.

### Association of diet with all-cause mortality

We utilized a fully adjusted Cox proportional hazards model that took into account demographic characteristics, sociological characteristics, personal lifestyle, and chronic disease counts to examine the hazard ratios (HRs) for all-cause mortality associated with various dietary patterns. Table 3 presents the results of our fully adjusted model, indicating that individuals in the top quintiles of PDI (HR_PDI_ = 0.81; 95% CI: 0.71, 0.90), hPDI (HR_hPDI_ = 0.79; 95% CI: 0.69, 0.93), and HFP (HR_HFP_ = 0.81; 95% CI: 0.70, 0.94) displayed a significant reduction in the risk of mortality compared with those in the lowest quintiles. In contrast, individuals in the top quintile of AF (HR_AF_ = 1.17; 95% CI: 1.00, 1.36) exhibited a significant increase in the risk of mortality compared with those in the lowest quintile. In our postimputation model (Supplemental Table 6), we also observed that individuals in the top quintiles of PDI (HR = 0.79; 95% CI: 0.70, 0.90), hPDI (HR = 0.82; 95% CI: 0.73, 0.93), and HPF (HR = 0.79; 95% CI: 0.68, 0.90) had a significantly lower risk of mortality than those in the lowest quintiles. However, a different conclusion arose with respect to individuals in the top quintile of AF (HR = 1.14; 95% CI: 0.99, 1.31; *P* = 0.077), who exhibited a significant increase in the risk of mortality compared with those in the lowest quintile.

**TABLE 3:** Results of survival analysis models for the relationship between each dietary pattern score and all-cause mortality

Population	Case, No.	Incidence Rate, per 1000 person-years	HR (95% CI)			
			Model 1	Model 2	Model 3	Model 4
PDI						
Q1	599	410.00	Ref.	Ref.	Ref.	Ref.
Q2	553	383.72	0.93 (0.83, 1.05)	0.93 (0.83, 1.05)	0.93 (0.82, 1.05)	0.90 (0.79, 1.02)
Q3	486	380.26	0.93 (0.82, 1.04)	0.93 (0.82, 1.04)	0.91 (0.80, 1.03)	0.88 (0.77, 1.00)
Q4	425	361.35	0.87 (0.77, 0.99)^1^	0.87 (0.77, 0.99)^1^	0.90 (0.79, 1.03)	0.87 (0.76, 1.00)^1^
Q5	422	338.36	0.81 (0.72, 0.92)^2^	0.82 (0.72, 0.92)^2^	0.83 (0.73, 0.94)^2^	0.81 (0.71, 0.90)^2^
hPDI						
Q1	613	418.29	Ref.	Ref.	Ref.	Ref.
Q2	478	394.20	0.94 (0.83, 1.06)	0.94 (0.83, 1.06)	0.92 (0.81, 1.04)	0.90 (0.79, 1.02)
Q3	513	368.61	0.87 (0.78, 0.98)^1^	0.87 (0.77, 0.98)^1^	0.84 (0.75, 0.95)^2^	0.82 (0.73, 0.93)^2^
Q4	447	343.06	0.81 (0.72, 0.92)^2^	0.81 (0.71, 0.91)^2^	0.80 (0.70, 0.91)^3^	0.78 (0.68, 0.89)^3^
Q5	434	352.70	0.83 (0.74, 0.94)^2^	0.83 (0.73, 0.94)^2^	0.81 (0.72, 0.93)^2^	0.79 (0.69, 0.93)^2^
uPDI						
Q1	521	365.80	Ref.	Ref.	Ref.	Ref.
Q2	573	367.59	1.00 (0.89, 1.13)	1.00 (0.87, 1.13)	0.97 (0.86, 1.10)	0.97 (0.85, 1.10)
Q3	478	370.73	1.02 (0.90, 1.15)	1.01 (0.89, 1.15)	1.03 (0.90, 1.18)	1.03 (0.90, 1.17)
Q4	501	386.72	1.06 (0.94, 1.20)	1.05 (0.93, 1.19)	1.07 (0.94, 1.22)	1.08 (0.94, 1.23)
Q5	412	397.89	1.10 (0.96, 1.25)	1.09 (0.96, 1.25)	1.09 (0.95, 1.26)	1.10 (0.95, 1.27)
HPF						
Q1	521	410.30	Ref.	Ref.	Ref.	Ref.
Q2	530	373.83	0.90 (0.80, 1.02)	0.89 (0.79, 1.00)	0.87 (0.77, 0.99)^1^	0.86 (0.75, 0.98)^1^
Q3	608	379.82	0.92 (0.82, 1.03)	0.91 (0.81, 1.02)	0.88 (0.79, 1.00)	0.87 (0.76, 0.99)^1^
Q4	472	374.20	0.91 (0.80, 1.03)	0.91 (0.80, 1.03)	0.89 (0.78, 1.02)	0.87 (0.75, 0.99)^1^
Q5	354	335.98	0.81 (0.71, 0.92)^2^	0.80 (0.70, 0.92)^2^	0.81 (0.70, 0.94)^2^	0.81 (0.70, 0.94)^2^
uHPF						
Q1	510	399.86	Ref.	Ref.	Ref.	Ref.
Q2	513	354.16	0.87 (0.77, 0.99)^1^	0.88 (0.78, 1.00)^1^	0.88 (0.77, 1.00)^1^	0.89 (0.78, 1.01)
Q3	910	388.98	0.97 (0.87, 1.08)	0.98 (0.88, 1.09)	0.99 (0.88, 1.11)	1.00 (0.89, 1.13)
Q4	177	348.21	0.86 (0.73, 1.02)	0.86 (0.73, 1.03)	0.89 (0.75, 1.06)	0.89 (0.75, 1.07)
Q5	375	363.47	0.90 (0.79, 1.03)	0.90 (0.79, 1.03)	0.94 (0.82, 1.08)	0.95 (0.82, 1.10)
AF						
Q1	510	376.42	Ref.	Ref.	Ref.	Ref.
Q2	659	355.58	0.94 (0.84, 1.06)	0.94 (0.84, 1.05)	0.92 (0.82, 1.04)	0.91 (0.80, 1.03)
Q3	515	361.06	0.96 (0.85, 1.08)	0.96 (0.85, 1.09)	0.95 (0.83, 1.08)	0.95 (0.83, 1.09)
Q4	448	402.01	1.07 (0.94, 1.22)	1.07 (0.94, 1.22)	1.07 (0.94, 1.23)	1.08 (0.94, 1.24)
Q5	353	413.14	1.10 (0.96, 1.26)	1.11 (0.97, 1.27)	1.15 (0.99, 1.33)	1.17 (1.00, 1.36)∗

model 1: unadjusted; model 2: adjusted for age and gender; model 3: adjusted for age, gender, residence, ethnic group, BMI, years of schooling, marital status, income status, current smoker, current alcohol drinker and regular exercise; model 4: adjusted for age, gender, residence, ethnic group, BMI, years of schooling, marital status, income status, current smoker, current alcohol drinker, regular exercise, and number of chronic diseases.

Abbreviations: PDI, overall plant-based diet index; hPDI, healthy plant-based diet index; uPDI, unhealthy plant-based diet index; HPF, healthy plant foods index; uHPF, unhealthy foods index; AF, animal foods index; Ref., Reference.

1 *P* < 0.05

2 *P* < 0.01

3 *P* < 0.001.

**FIGURE 3:**
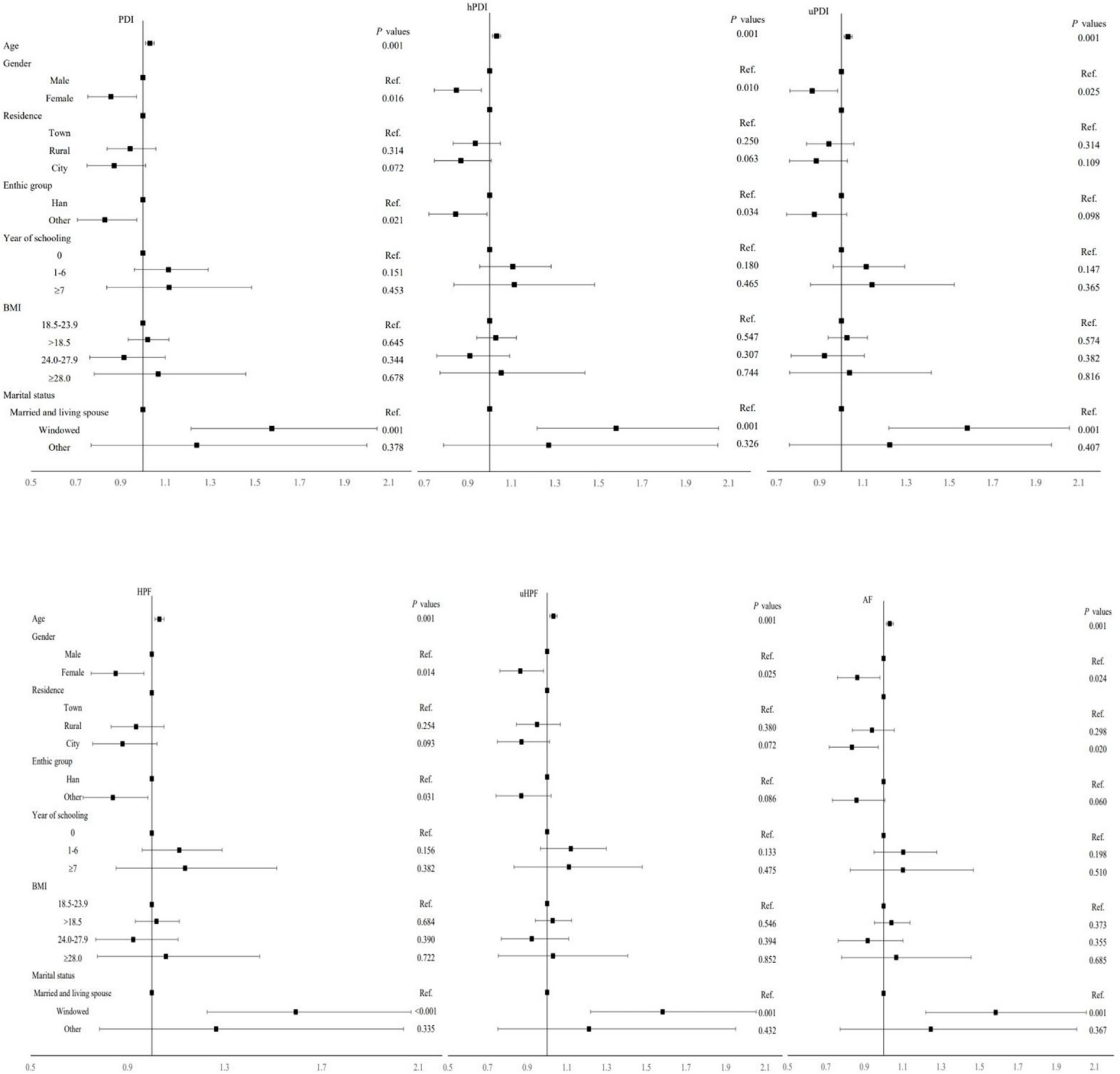
HR values for demographic characteristics of each dietary pattern.

A forest plot (Figure 3) of the HR values for age, gender, place of residence, ethnicity, years of schooling, BMI, and marital status has been generated. Across the different dietary patterns, we have observed that the mortality risk is higher for individuals of advanced age and for widowed centenarians. In contrast, females and non-Han ethnicities exhibit a lower mortality risk.

## Discussion

Within this Chinese centenarian national prospective cohort study, we have observed significant associations between PDI, hPDI, HPF, and reduced risks of chronic disease incidence and mortality. Nevertheless, higher AF scores may give rise to an increased risk of mortality.

Our research findings on centenarians are similar to a study on Chinese individuals aged ≥ y65 [22]; in this study, the overall plant-based dietary pattern reduced the mortality risk by 8% (HR PDI Q5 compared with Q1 = 0.92; 95% CI: 0.86, 0.98), whereas the high-scoring healthy plant-based dietary pattern reduced the mortality risk by 19%, which is consistent with previous literature (HR PDI Q5 compared with Q1 = 0.81; 95% CI: 0.76, 0.87). Our study also showed that an unhealthy plant-based diet increased the mortality risk by 17% (HR uPDI Q5 compared with Q1 = 1.17; 95% CI: 1.09, 1.26). Similarly, a study on United Kingdom adults with an average age of 56.1 y showed that a high-hPDI score reduced the mortality risk by 16% (HR PDI Q4 compared with Q1 = 0.84; 95% CI: 0.78, 0.91), whereas an unhealthy plant-based diet increased the mortality risk by 23% (HR uPDI Q4 compared with Q1 = 1.23; 95% CI: 1.14, 1.32) [30]. Another study on United States adults with an average age of 52 y showed that a healthy plant-based diet decreased the mortality risk by 14% [31]. According to a data-based study involving Spanish adults aged ≥18 y, the consumption of a healthy plant-based diet was associated with a 14% reduction in the mortality risk (HR = 0.86; 95% CI: 0.74, 0.99), but all mortality risk of an unhealthy plant-based diet did not reach statistical significance [32]. Our findings concur with studies done in American-wide population [33]. Apart from this, our evaluation of the animal-based foods (AF) dietary pattern score revealed a link between adverse health outcomes and centenarians who consumed such a diet, with nonsignificant trends being observed in an unhealthy plant-based dietary pattern. The unique dietary characteristics of Chinese centenarians may account for the lack of statistical significance observed in this regard.

Pervasive experimental evidence has demonstrated that, in general, a plant-based diet is beneficial to health, largely because of its alignment with global dietary recommendations for chronic disease prevention and management [17,22,[34], [35], [36], [37], [38], [39]]. Such a regimen can enhance metabolic regulation, inflammation resistance, and oxidation processes through the consumption of a nourishing plant-based diet replete with fiber-rich and high-quality protein-filled food sources, thereby contributing to the reduction of chronic illnesses [[40], [41], [42], [43], [44], [45]]. Nonetheless, it is worth mentioning that the centenarian population mainly comprises a relatively healthy elderly cohort that can live up to the age of 100 y because of the possible fortification of their immune systems and resilience [6,10,46]. It is noteworthy that the incidence rate of chronic diseases tends to be lower in this group [47]. Thus, our observational findings suggest that despite the fact that a high score for both whole plant-based dietary patterns and healthy eating practices can reduce the mortality risk of centenarians, not all wholesome plant-based foods exert this effect [[48], [49], [50]].

The primary advantage of our study lies in its prospective design, featuring the world’s largest sample size of centenarians. Despite its advantages, our study has some limitations. First, China’s vast population means that our sample size only represents a fraction of centenarians, and many others have not been considered. Second, we used a simplified food-frequency questionnaire to evaluate dietary intake, which, despite validation among Asians, remains subjective, limiting the evaluation tool. In addition, we included only limited confounding factors, and it is probable that controlled variable results through confounding variable control might have some errors. Third, we evaluated a limited number of foods, only 16, when there are several other foods that Chinese people often consume, such as sweet potatoes, potatoes, peppers, along with various local specialties, limiting the evaluation of food types. Furthermore, >80% of participants were female and >93% were of a single ethnic population, substantially limiting the generalizability of the results. Finally, our study is limited to centenarians in China and may not be generalizable to the centenarians of other countries.

In conclusion, despite the fact that a higher score in both a predominantly plant-based dietary pattern and a healthy dietary pattern can decrease the risk of death among centenarians, not all HPF have this effect. Higher AF predicted a higher risk of mortality, whereas higher PDI, hPDI, and HPF were associated with a lower risk of mortality. Our findings address the lack of long-term tracking of dietary habits and health outcomes among Chinese centenarians and offer a unique approach for the study of centenarians worldwide.

## Authors’ contributions

The authors’ responsibilities were as follows – LY: designed the study; LY, ZZ: controlled the quality of the data and performed statistical analysis; LY, QJ, YL, JS: managed and checked all the data; JS, YQ, LY: contributed to manuscript preparation, editing, and review; and all authors: read, checked, and approved the final manuscript.

## Conflict of interest

The authors report no conflicts of interest.

## Data Availability

Data described in the manuscript, code book, and analytic code will be made available upon application and approval.

## Appendix A Supplementary data

Multimedia component1 Multimedia component1
