# Supplementary material for: Association between Plant-based Diet and Risk of Chronic Diseases and All-Cause Mortality in Centenarians in China: A Cohort Study
Source: Curr Dev Nutr. 2023 Dec 19;8(1):102065. doi: 10.1016/j.cdnut.2023.102065 (PMC10792746; doi:10.1016/j.cdnut.2023.102065)
Supplement: Multimedia component1 [file mmc1.docx]

**Supplementary Material**

**Association between plant-based diet and risk of chronic diseases and all-cause mortality in centenarians in China: A cohort study**

Lei Yuan^1†*^, QinQin Jiang^1†^, Yinghong Zhai^2†^, Zhe Zhao^1†^, Yijun Liu^1^, Fangyuan Hu^3*^, Yi Qian^4*^, Jinhai Sun^1*^

1.Department of Health Management, Faculty of Military Health Service, Naval Medical University, Shanghai, China

2. Clinical Research Unit, School of Medicine, Shanghai 9th People's Hospital Affiliated to Shanghai JiaoTong University, Shanghai 200011, China.

3. Department of Medical Service, Naval Hospital of Eastern Theater, Zhoushan, China

4. College of Health Management, Southern Medical University, Guangzhou, China

^†^These authors contributed equally as co-first authors.

^*^These authors contributed equally as co-corresponding authors

**Corresponding author:** Lei Yuan

**Affiliation:** Department of Health Management, Faculty of Military Health Service, Naval Medical University, Shanghai, China

**Address:** No. 800 Xiangyin Road, Shanghai 200433, PR.China

**Tel:** +86-021-81871404; Fax: +86-021-81871404;

**E-mail:** [yuanleigz@163.com](mailto:yuanleigz@163.com)

**Method section1**

The Calculations of HPFs, LPFs, and AFs for Each Participant were Computed Using the Following Formulas:

HPFs=HPF/4

uHPFs=uHPF/1.5

AFs=AF/2.5

**Method section 2**

***Covariables***

***Demographic characteristics***

Age was measured as a continuous variable. Gender was classified into female and male. Residence was categorized as urban, rural or town. Ethnicity was divided into Han and other ethnic groups. BMI was calculated as weight in kilograms divided by height in meters squared, and was classified into four categories as follows: <18.5, 18.5-23.9, 24.0-27.9, and >28.0.

***Sociological characteristics***

Years of schooling was classiﬁed according to time in school as 0 years, 1–6 years, and ≥ 7 years. Marital status was classified married and living with spouse, widowed, and others (including married but not living with a spouse, divorced, and never married). Income status included general, poor and rich according to the answer to the question ‘How do you rate your economic status compared with other local people?’

***Personal lifestyle***

Based on participants’ responses to the questions ‘Do you smoke or not at the present time?’, ‘Do you drink or not at present??’ and ‘Do you do exercises regularly or not at present?’, current smoker, current alcohol drinker, and regular exercise were categorized as yes and no.

In the longitudinal analysis, the included covariates were divided into four categories: demographic characteristics, sociological characteristics, personal lifestyle, and health status. The first three categories were consistent with the classification used in the cross-sectional survey assessment. Health status was based on the 9 types of chronic diseases, which were the primary outcome variables of the cross-sectional analysis.

Table S1 lists the allocation of differential consumption frequency of 16 food types across various dietary patterns

| Items | Frequency | PDI | hPDI | uPDI | HPF | uHPF | AF |
| --- | --- | --- | --- | --- | --- | --- | --- |
| Plant foods (healthy) |  |  |  |  |  |  |  |
| Whole grains | Not | 1 | 1 | 5 | 1 | - | - |
|  | As staple food | 5 | 5 | 1 | 5 | - | - |
| Vegetable oils | Not | 1 | 1 | 5 | 1 | - | - |
|  | As main cooking grease | 5 | 5 | 1 | 5 | - | - |
| Fresh fruits | Rarely or never | 1 | 1 | 5 | 1 | - | - |
|  | Occasionally | 2 | 2 | 4 | 2 | - | - |
|  | Quite often | 4 | 4 | 2 | 4 | - | - |
|  | Almost everyday | 5 | 5 | 1 | 5 | - | - |
| Fresh vegetables | Rarely or never | 1 | 1 | 5 | 1 | - | - |
|  | Occasionally | 2 | 2 | 4 | 2 | - | - |
|  | Quite often | 4 | 4 | 2 | 4 | - | - |
|  | Almost everyday | 5 | 5 | 1 | 5 | - | - |
| Legumes | Rarely or never | 1 | 1 | 5 | 1 | - | - |
|  | Occasionally | 2 | 2 | 4 | 2 | - | - |
|  | ≥1 time per month | 3 | 3 | 3 | 3 | - | - |
|  | ≥1 time per work | 4 | 4 | 2 | 4 | - | - |
|  | Almost everyday | 5 | 5 | 1 | 5 | - | - |
| Garlic | Rarely or never | 1 | 1 | 5 | 1 | - | - |
|  | Occasionally | 2 | 2 | 4 | 2 | - | - |
|  | ≥1 time per month | 3 | 3 | 3 | 3 | - | - |
|  | ≥1 time per work | 4 | 4 | 2 | 4 | - | - |
|  | Almost everyday | 5 | 5 | 1 | 5 | - | - |
| Nuts | Rarely or never | 1 | 1 | 5 | 1 | - | - |
|  | Occasionally | 2 | 2 | 4 | 2 | - | - |
|  | ≥1 time per month | 3 | 3 | 3 | 3 | - | - |
|  | ≥1 time per work | 4 | 4 | 2 | 4 | - | - |
|  | Almost everyday | 5 | 5 | 1 | 5 | - | - |
| Tea | Rarely or never | 1 | 1 | 5 | 1 | - | - |
|  | Occasionally | 2 | 2 | 4 | 2 | - | - |
|  | ≥1 time per month | 3 | 3 | 3 | 3 | - | - |
|  | ≥1 time per work | 4 | 4 | 2 | 4 | - | - |
|  | Almost everyday | 5 | 5 | 1 | 5 | - | - |
| Plant foods (unhealthy) |  |  |  |  |  |  |  |
| Refined grains | Not | 1 | 5 | 1 | - | 5 | - |
|  | As main cooking grease | 5 | 1 | 5 | - | 1 | - |
| Preserved vegetables | Rarely or never | 1 | 5 | 1 | - | 5 | - |
|  | Occasionally | 2 | 4 | 2 | - | 4 | - |
|  | ≥1 time per month | 3 | 3 | 3 | - | 3 | - |
|  | ≥1 time per work | 4 | 2 | 4 | - | 2 | - |
|  | Almost everyday | 5 | 1 | 5 | - | 1 | - |
| Sugar | Rarely or never | 1 | 5 | 1 | - | 5 | - |
|  | Occasionally | 2 | 4 | 2 | - | 4 | - |
|  | ≥1 time per month | 3 | 3 | 3 | - | 3 | - |
|  | ≥1 time per work | 4 | 2 | 4 | - | 2 | - |
|  | Almost everyday | 5 | 1 | 5 | - | 1 | - |
| Animal foods |  |  |  |  |  |  |  |
| Meat | Rarely or never | 5 | 5 | 5 | - | - | 1 |
|  | Occasionally | 4 | 4 | 4 | - | - | 2 |
|  | ≥1 time per month | 3 | 3 | 3 | - | - | 3 |
|  | ≥1 time per work | 2 | 2 | 2 | - | - | 4 |
|  | Almost everyday | 1 | 1 | 1 | - | - | 5 |
| Fish and aquatic products | Rarely or never | 5 | 5 | 5 | - | - | 1 |
|  | Occasionally | 4 | 4 | 4 | - | - | 2 |
|  | ≥1 time per month | 3 | 3 | 3 | - | - | 3 |
|  | ≥1 time per work | 2 | 2 | 2 | - | - | 4 |
|  | Almost everyday | 1 | 1 | 1 | - | - | 5 |
| Eggs | Rarely or never | 5 | 5 | 5 | - | - | 1 |
|  | Occasionally | 4 | 4 | 4 | - | - | 2 |
|  | ≥1 time per month | 3 | 3 | 3 | - | - | 3 |
|  | ≥1 time per work | 2 | 2 | 2 | - | - | 4 |
|  | Almost everyday | 1 | 1 | 1 | - | - | 5 |
| Milk or dairy products | Rarely or never | 5 | 5 | 5 | - | - | 1 |
|  | Occasionally | 4 | 4 | 4 | - | - | 2 |
|  | ≥1 time per month | 3 | 3 | 3 | - | - | 3 |
|  | ≥1 time per work | 2 | 2 | 2 | - | - | 4 |
|  | Almost everyday | 1 | 1 | 1 | - | - | 5 |
| Animal fat | No | 5 | 5 | 5 | - | - | 1 |
|  | Yes | 1 | 1 | 1 | - | - | 5 |

Abbreviations: PDI, overall plant-based diet index; hPDI, healthful plant-based diet index; uPDI, unhealthful plant-based diet index; HPF, healthy plant food index; uHPF, unhealthy food index; AF, animal food index.

-: Not included in the calculation

Table S2 displays the baseline characteristics of the cohort participants categorized by quintiles of PDI

| Variables | Overall | PDI Q1 | PDI Q2 | PDI Q3 | PDI Q4 | PDI Q5 |
| --- | --- | --- | --- | --- | --- | --- |
| Age(years), mean(s.d.) | 102.33 (0.05) | 102.50 (0.08) | 102.38 (0.10) | 102.23 (0.09) | 102.12 (0.12) | 102.23 (0.14) |
| Gender |  |  |  |  |  |  |
| Male | 504(18.84) | 99(15.74) | 105(17.59) | 101(19.46) | 94(20.22) | 105(22.58) |
| Female | 2171(81.16) | 530(84.26) | 492(82.41) | 418(80.54) | 371(79.78) | 360(77.42) |
| Residence |  |  |  |  |  |  |
| Town | 427(15.96) | 91(14.47) | 86(14.41) | 79(15.22) | 79(16.99) | 92(19.78) |
| Rural | 1773(66.28) | 463(73.61) | 392(65.66) | 343(66.09) | 289(62.15) | 286(61.51) |
| City | 475(17.76) | 75(11.92) | 119(19.93) | 97(18.69) | 97(20.86) | 87(18.71) |
| Ethnic group |  |  |  |  |  |  |
| Han | 2471(92.37) | 554(88.08) | 560(93.80) | 489(94.22) | 439(94.41) | 429(92.26) |
| Other | 204(7.63) | 75(11.92) | 37(6.20) | 30(5.78) | 26(5.59) | 36240(7793.55) |
| BMI (kg/m^2^) |  |  |  |  |  |  |
| 18.5-23.9 | 1156(43.21) | 240(38.16) | 258(43.22) | 233(44.89) | 217(46.67) | 208(44.73) |
| <18.5 | 1159(43.33) | 304(48.33) | 258(43.22) | 202(38.92) | 193(41.51) | 202(43.44) |
| 24.0-27.9 | 140(5.23) | 31(4.93) | 29(4.86) | 37(7.13) | 22(4.73) | 21(4.52) |
| ≥28.0 | 46(1.72) | 6(0.95) | 7(1.17) | 7(1.35) | 9(1.94) | 17(3.66) |
| Missing | 174(6.50) | 48(7.63) | 45(7.54) | 40(7.71) | 24(5.16) | 17(3.66) |
| Year of schooling |  |  |  |  |  |  |
| 0 | 2308(86.28) | 554(88.08) | 507(84.92) | 452(87.09) | 403(86.67) | 392(84.30) |
| 1-6 | 288(10.77) | 58(9.22) | 68(11.39) | 52(10.02) | 47(10.11) | 63(13.55) |
| ≥7 | 67(2.50) | 16(2.54) | 17(2.85) | 12(2.31) | 14(3.01) | 8(1.72) |
| Missing | 12(0.45) | 1(0.16) | 5(0.84) | 3(0.58) | 1(0.22) | 2(0.43) |
| Marital status |  |  |  |  |  |  |
| Married and living spouse | 86(3.21) | 14(2.23) | 17(2.85) | 17(3.28) | 20(4.30) | 18(3.87) |
| Windowed | 2561(95.74) | 614(97.62) | 576(96.48) | 495(95.38) | 432(92.90) | 444(95.48) |
| Other | 28(1.05) | 1(0.16) | 4(0.67) | 7(1.35) | 13(2.80) | 3(0.65) |
| Income status |  |  |  |  |  |  |
| General | 1833(68.52) | 425(67.57) | 406(68.01) | 373(71.87) | 313(67.31) | 316(67.96) |
| Rich | 326(12.19) | 68(10.81) | 61(10.22) | 66(12.72) | 60(12.90) | 71(15.27) |
| Poor | 507(18.95) | 133(21.14) | 128(21.44) | 77(14.84) | 91(19.57) | 78(16.77) |
| Missing | 9(0.34) | 3(0.48) | 2(0.34) | 3(0.58) | 1(0.22) | 0(0.00) |
| Current Smoker |  |  |  |  |  |  |
| No | 2478(92.64) | 586(93.16) | 561(93.97) | 488(94.03) | 418(89.89) | 425(91.40) |
| Yes | 197(7.36) | 43(6.84) | 36(6.03) | 31(5.97) | 47(10.11) | 40(8.60) |
| Current alcohol drinker |  |  |  |  |  |  |
| No | 2330(87.10) | 548(87.12) | 524(87.77) | 459(88.44) | 414(89.03) | 385(82.80) |
| Yes | 345(12.90) | 81(12.88) | 73(12.23) | 60(11.56) | 51(10.97) | 80(17.20) |
| Regular exercise |  |  |  |  |  |  |
| No | 2254(84.26) | 529(84.10) | 518(86.77) | 448(86.32) | 377(81.08) | 382(82.15) |
| Yes | 421(15.74) | 100(15.90) | 79(13.23) | 71(13.68) | 88(18.92) | 83(17.85) |
| Hypertension |  |  |  |  |  |  |
| No | 2325(86.92) | 554(88.08) | 513(85.93) | 453(87.28) | 399(85.81) | 406(87.31) |
| Yes | 291(10.88) | 51(8.11) | 72(12.06) | 59(11.37) | 57(12.26) | 52(11.18) |
| Missing | 59(2.21) | 24(3.82) | 12(2.01) | 7(1.35) | 9(1.94) | 7(1.51) |
| Diabetes |  |  |  |  |  |  |
| No | 2615(97.76) | 599(95.23) | 589(98.66) | 511(98.46) | 458(98.49) | 458(98.49) |
| Yes | 13(0.49) | 6(0.95) | 3(0.50) | 2(0.39) | 1(0.22) | 1(0.22) |
| Missing | 47(1.76) | 24(3.82) | 5(0.84) | 6(1.16) | 6(1.29) | 6(1.29) |
| Heart disease |  |  |  |  |  |  |
| No | 2463(92.07) | 583(92.69) | 550(92.13) | 480(92.49) | 431(92.69) | 419(90.11) |
| Yes | 164(6.13) | 24(3.82) | 41(6.87) | 33(6.36) | 28(6.02) | 38(8.17) |
| Missing | 48(1.79) | 22(3.50) | 6(1.01) | 6(1.16) | 6(1.29) | 8(1.72) |
| Stroke or CVD |  |  |  |  |  |  |
| No | 2544(95.10) | 594(94.44) | 559(93.63) | 497(95.76) | 446(95.91) | 448(96.34) |
| Yes | 94(3.51) | 19(3.02) | 33(5.53) | 17(3.28) | 15(3.23) | 10(2.15) |
| Missing | 37(1.38) | 16(2.54) | 5(0.84) | 5(0.96) | 4(0.86) | 7(1.51) |
| Respiratory disease |  |  |  |  |  |  |
| No | 2396(89.57) | 555(88.24) | 533(89.28) | 460(88.63) | 424(91.18) | 424(91.18) |
| Yes | 247(9.23) | 60(9.54) | 60(10.05) | 55(10.60) | 36(7.74) | 36(7.74) |
| Missing | 32(1.20) | 14(2.23) | 4(0.67) | 4(0.77) | 5(1.08) | 5(1.08) |
| Gastric or duodenal ulcer |  |  |  |  |  |  |
| No | 2523(94.32) | 583(92.69) | 569(95.31) | 491(94.61) | 440(94.62) | 440(94.62) |
| Yes | 105(3.93) | 27(4.29) | 19(3.18) | 24(4.62) | 17(3.66) | 18(3.87) |
| Missing | 47(1.76) | 19(3.02) | 9(1.51) | 4(0.77) | 8(1.72) | 7(1.51) |
| Arthritis |  |  |  |  |  |  |
| No | 2165(80.93) | 475(75.52) | 475(79.56) | 436(84.01) | 390(83.87) | 389(83.66) |
| Yes | 478(17.87) | 144(22.89) | 116(19.43) | 78(15.03) | 69(14.84) | 71(15.27) |
| Missing | 32(1.20) | 10(1.59) | 6(1.01) | 5(0.96) | 6(1.29) | 5(1.08) |
| Dementia |  |  |  |  |  |  |
| No | 2531(94.62) | 579(92.05) | 568(95.14) | 495(95.38) | 443(95.27) | 443(95.27) |
| Yes | 112(4.19) | 35(5.56) | 24(4.02) | 22(4.24) | 15(3.23) | 16(3.44) |
| Missing | 32(1.20) | 15(2.38) | 5(0.84) | 2(0.39) | 4(0.86) | 6(1.29) |
| Cholecystitis or cholelith disease |  |  |  |  |  |  |
| No | 2588(96.75) | 602(95.71) | 580(97.15) | 503(96.92) | 449(96.56) | 454(97.63) |
| Yes | 42(1.57) | 8(1.27) | 7(1.17) | 11(2.12) | 11(2.37) | 5(1.08) |
| Missing | 45(1.68) | 19(3.02) | 19(3.18) | 5(0.96) | 5(1.08) | 6(1.29) |
| Number of chronic diseases |  |  |  |  |  |  |
| 0 | 1537(57.46) | 346(55.01) | 326(54.61) | 302(58.19) | 278(59.78) | 285(61.29) |
| 1 | 717(26.80) | 171(27.19) | 165(27.64) | 141(27.17) | 119(25.59) | 121(26.02) |
| ≥2 | 314(11.74) | 72(11.45) | 80(13.40) | 62(11.95) | 52(11.18) | 48(10.32) |
| Missing | 107(4.00) | 40(6.36) | 26(4.36) | 14(2.70) | 16(3.44) | 11(2.37) |

Table S3 Presents the scores of various dietary patterns among centenarian participants

| Dietary | Cross-sectional | | Cohort | |
| --- | --- | --- | --- | --- |
|  | Mean | SD | Mean | SD |
| PDI | 46.95 | 6.29 | 46.92 | 6.09 |
| hPDI | 44.43 | 5.76 | 44.32 | 5.68 |
| uPDI | 51.09 | 6.26 | 51.30 | 6.26 |
| HPF | 21.63 | 4.79 | 21.48 | 4.81 |
| uHPF | 9.91 | 2.41 | 9.96 | 2.42 |
| AF | 14.59 | 3.58 | 14.53 | 3.61 |
| HPFs | 5.41 | 1.20 | 5.37 | 1.02 |
| uHPFs | 6.61 | 1.60 | 6.64 | 1.61 |
| AFs | 5.84 | 1.43 | 5.81 | 1.45 |

Table S4 Displays the scores and rankings of various food items among centenarian participants in a cross-sectional study

| Food | Mean | Std | Order in total | Order in HPF | Order in uHPF | Order in AF |
| --- | --- | --- | --- | --- | --- | --- |
| Whole grains | 0.17 | 0.80 | 16 | 8 | - | - |
| Vegetable oils | 4.37 | 1.45 | 2 | 1 | - | - |
| Fresh fruits | 2.66 | 1.46 | 9 | 4 | - | - |
| Fresh vegetables | 4.16 | 1.19 | 3 | 2 | - | - |
| Legumes | 3.37 | 1.29 | 6 | 3 | - | - |
| Garlic | 2.53 | 1.50 | 11 | 5 | - | - |
| Nuts | 1.42 | 0.92 | 15 | 7 | - | - |
| Tea | 1.97 | 1.59 | 13 | 6 | - | - |
| Refined grains | 4.83 | 0.80 | 1 | - | 1 | - |
| Preserved vegetables | 2.12 | 1.50 | 12 | - | 3 | - |
| Sugar | 2.96 | 1.59 | 7 | - | 2 | - |
| Meat | 3.66 | 1.31 | 5 | - | - | 2 |
| Fish | 2.77 | 1.34 | 8 | - | - | 3 |
| Eggs | 3.94 | 1.23 | 4 | - | - | 1 |
| Milk | 2.59 | 1.69 | 10 | - | - | 4 |
| Animal fat | 1.63 | 1.45 | 14 | - | - | 5 |

-: Not involved in the sorting.

Table S5 Regression Models with Missing Value Imputation Examining the Association between Dietary Pattern Scores and Chronic Disease Prevalence

| Population | β (95%CI) | | |
| --- | --- | --- | --- |
|  | Model 1 | Model 2 | Model 3 |
| Number of chronic diseases ^a^ |  |  |  |
| 0 | Ref. | Ref. | Ref. |
| 1 | -0.40 (-0.90,0.11) | -0.39 (-0.89,0.11) | -0.40 (-0.90,0.08) |
| ≥2 | -0.63 (-1.25,-0.01) * | -0.63 (-1.25,-0.02) * | -0.78 (-1.40,-0.15) * |
| Number of chronic diseases ^b^ |  |  |  |
| 0 | Ref. | Ref. | Ref. |
| 1 | -0.63 (-1.09,-0.17) ** | -0.61 (-1.07,-0.16) ** | -0.64 (-1.10,-0.18) ** |
| ≥2 | -0.31 (-0.90,0.28) | -0.34 (-0.92,0.25) | -0.55 (-1.15,0.04) |
| Number of chronic diseases ^c^ |  |  |  |
| 0 | Ref. | Ref. | Ref. |
| 1 | 0.31 (-0.18,0.80) | 0.27 (-0.22,0.76) | 0.28 (-0.18,0.74) |
| ≥2 | 0.26 (-0.38,0.91) | 0.32 (-0.32,0.96) | 0.51 (-0.10,1.13) |
| Number of chronic diseases ^d^ |  |  |  |
| 0 | Ref. | Ref. | Ref. |
| 1 | -0.36 (-0.75,0.02) | -0.34 (-0.73,0.04) | -0.36 (-0.73,0.02) |
| ≥2 | -0.43 (-0.92,0.06) | -0.46 (-0.95,0.03) | -0.64 (-1.12,-0.16) ** |
| Number of chronic diseases ^e^ |  |  |  |
| 0 | Ref. | Ref. | Ref. |
| 1 | 0.11 (-0.08,0.31) | 0.11 (-0.09,0.31) | 0.12 (-0.08,0.31) |
| ≥2 | -0.18 (-0.43,0.06) | -0.17 (-0.42,0.07) | -0.13 (-0.38,0.12) |
| Number of chronic diseases ^f^ |  |  |  |
| 0 | Ref. | Ref. | Ref. |
| 1 | 0.15 (-0.13,0.43) | 0.16 (-0.12,0.44) | 0.16 (-0.11,0.44) |
| ≥2 | 0.01 (-0.35,0.38) | -0.001 (-0.37,0.37) | 0.004 (-0.36,0.37) |

* *P*<0.05, ***P*<0.01.

a: the outcome is PDI, b: the outcome is hPDI, c: the outcome is uPDI, d: the outcome is HPF, e: the outcome is uHPF, f: the outcome is AF.

model 1: unadjusted; model 2: adjusted for age and gender; model 3: adjusted for age, gender, residence, ethnic group, BMI, years of schooling, marital status, income status, current smoker, current alcohol drinker and regular exercise.

Abbreviations: PDI, overall plant-based diet index; hPDI, healthful plant-based diet index; uPDI, unhealthful plant-based diet index; HPF, healthy plant food index; uHPF, unhealthy food index; AF, animal food index; Ref., Reference.

Table S6 Association between Each Dietary Pattern Score and All-Cause Mortality: Survival Analysis Model Results with Missing Value Imputation

| Population | HR^a^ | 95%CI of HR | *P* values |
| --- | --- | --- | --- |
| PDI |  |  |  |
| Q1 | Ref. | Ref. | Ref. |
| Q2 | 0.91 | (0.81,1.03) | 0.126 |
| Q3 | 0.90 | (0.80,1.03) | 0.122 |
| Q4 | 0.88 | (0.77,0.99) | 0.039 |
| Q5 | 0.79 | (0.70,0.90) | 0.001 |
| hPDI |  |  |  |
| Q1 | Ref. |  |  |
| Q2 | 0.92 | (0.82,1.05) | 0.224 |
| Q3 | 0.86 | (0.76,0.97) | 0.014 |
| Q4 | 0.79 | (0.70,0.90) | <0.001 |
| Q5 | 0.82 | (0.73,0.93) | 0.002 |
| uPDI |  |  |  |
| Q1 | Ref. |  |  |
| Q2 | 0.97 | (0.86,1.11) | 0.678 |
| Q3 | 1.00 | (0.88,1.14) | 1.000 |
| Q4 | 1.05 | (0.92,1.20) | 0.429 |
| Q5 | 1.07 | (0.94,1.23) | 0.287 |
| HPF |  |  |  |
| Q1 | Ref. |  |  |
| Q2 | 0.86 | (0.76,0.98) | 0.019 |
| Q3 | 0.88 | (0.77,1.01) | 0.030 |
| Q4 | 0.90 | (0.79,0.98) | 0.091 |
| Q5 | 0.79 | (0.68,0.90) | <0.001 |
| uHPF |  |  |  |
| Q1 | Ref. |  |  |
| Q2 | 0.87 | (0.76,0.98) | 0.024 |
| Q3 | 0.97 | (0.86,1.08) | 0.543 |
| Q4 | 0.86 | (0.73,1.02) | 0.085 |
| Q5 | 0.90 | (0.78,1.02) | 0.107 |
| AF |  |  |  |
| Q1 | Ref. |  |  |
| Q2 | 0.93 | (0.83,1.05) | 0.228 |
| Q3 | 0.98 | (0.86,1.11) | 0.722 |
| Q4 | 1.20 | (0.95,1.23) | 0.218 |
| Q5 | 1.14 | (0.99,1.31) | 0.077 |

a: adjusted for age, gender, residence, ethnic group, BMI, years of schooling, marital status, income status, current smoker, current alcohol drinker, regular exercise and number of chronic diseases.

Abbreviations: PDI, overall plant-based diet index; hPDI, healthful plant-based diet index; uPDI, unhealthful plant-based diet index; HPF, healthy plant food index; uHPF, unhealthy food index; AF, animal food index; Ref., reference.


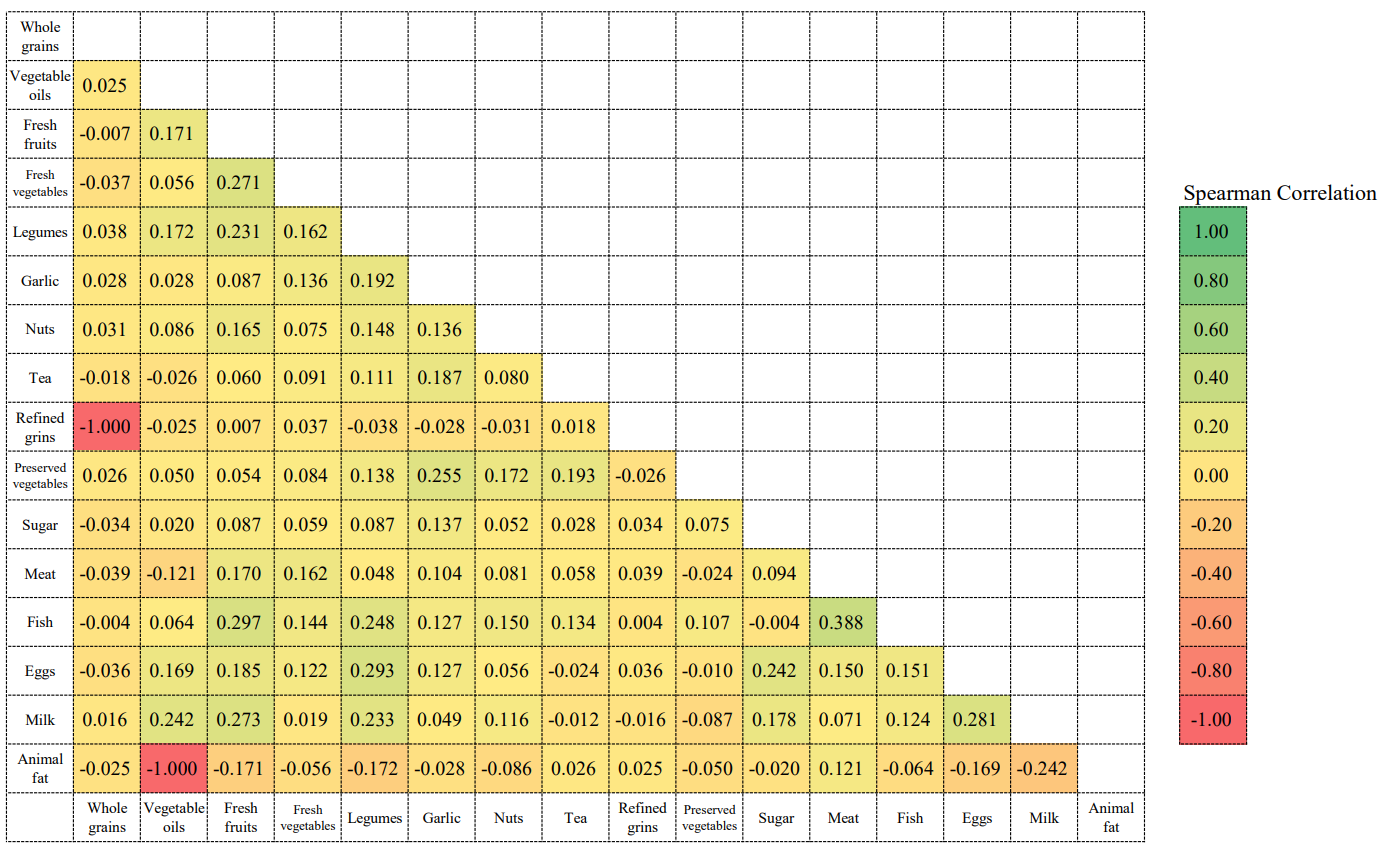
Figure S1 The pairwise Spearman correlation coefficient between different food groups of cross-sectional.


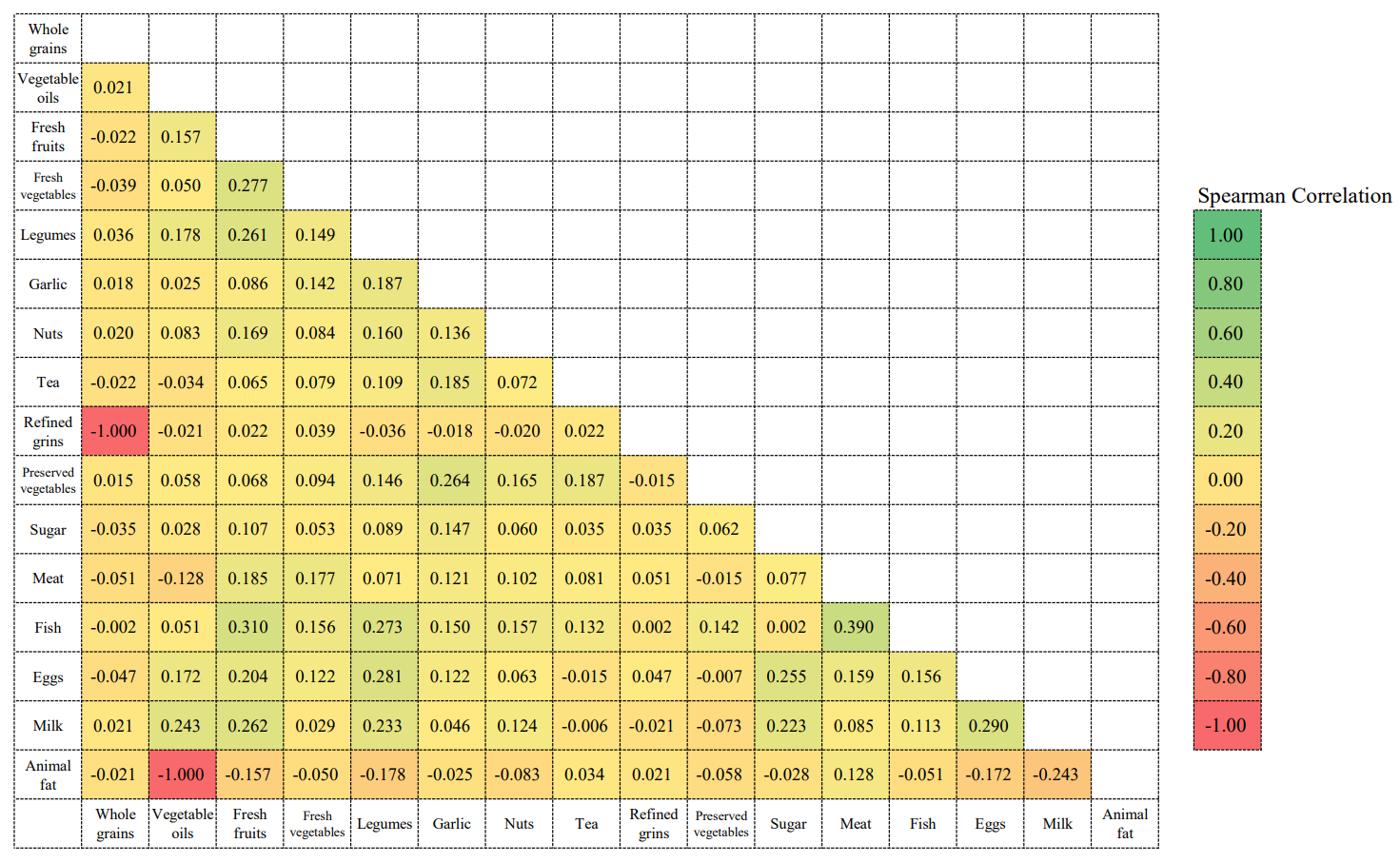
Figure S2 Pairwise Spearman Correlation Coefficients between Different Food Groups among Cohorts


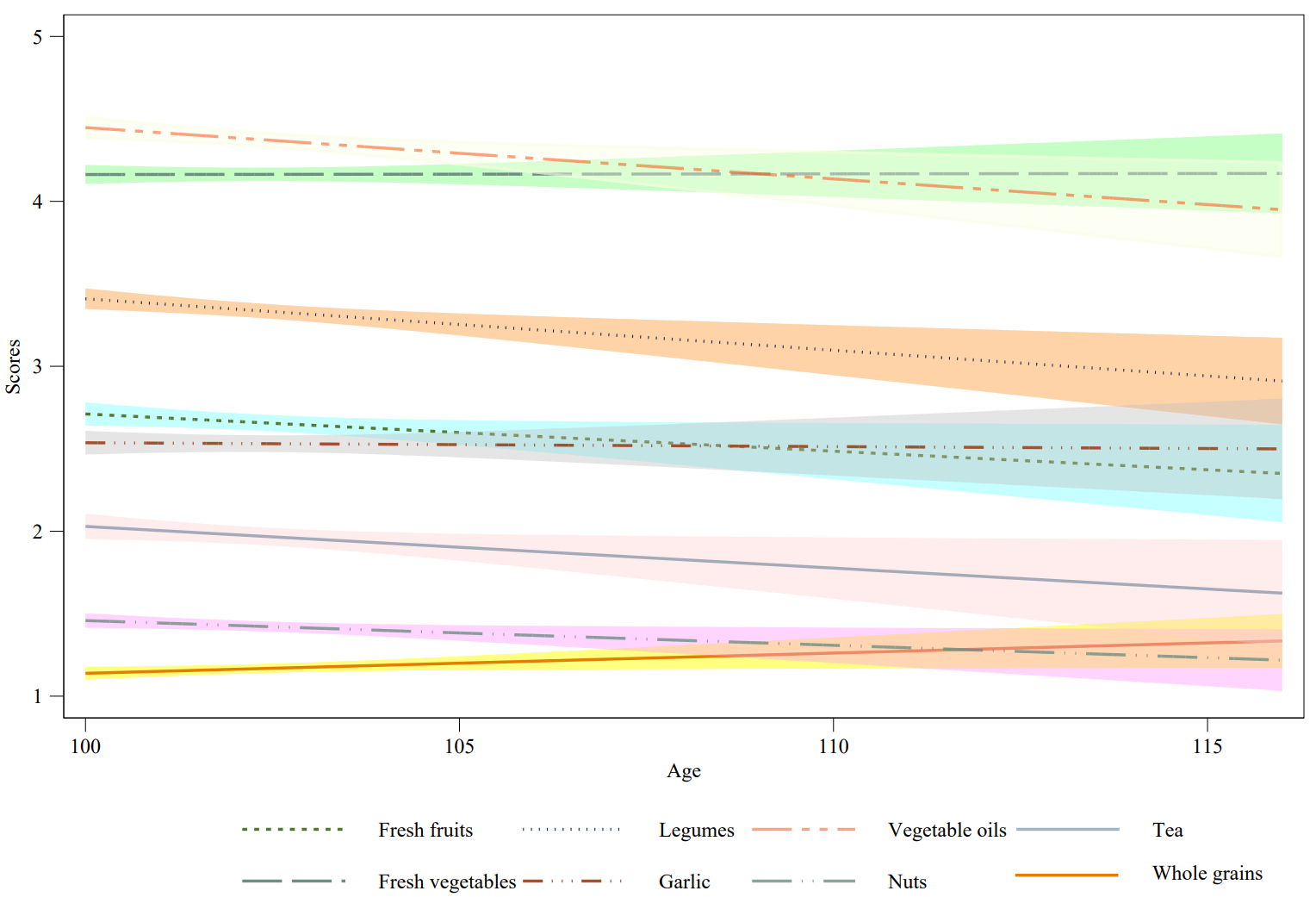
Figure S3 Distribution of Healthy Plant Food Scores among Chinese Centenarians in a Cross-Sectional Survey by Age


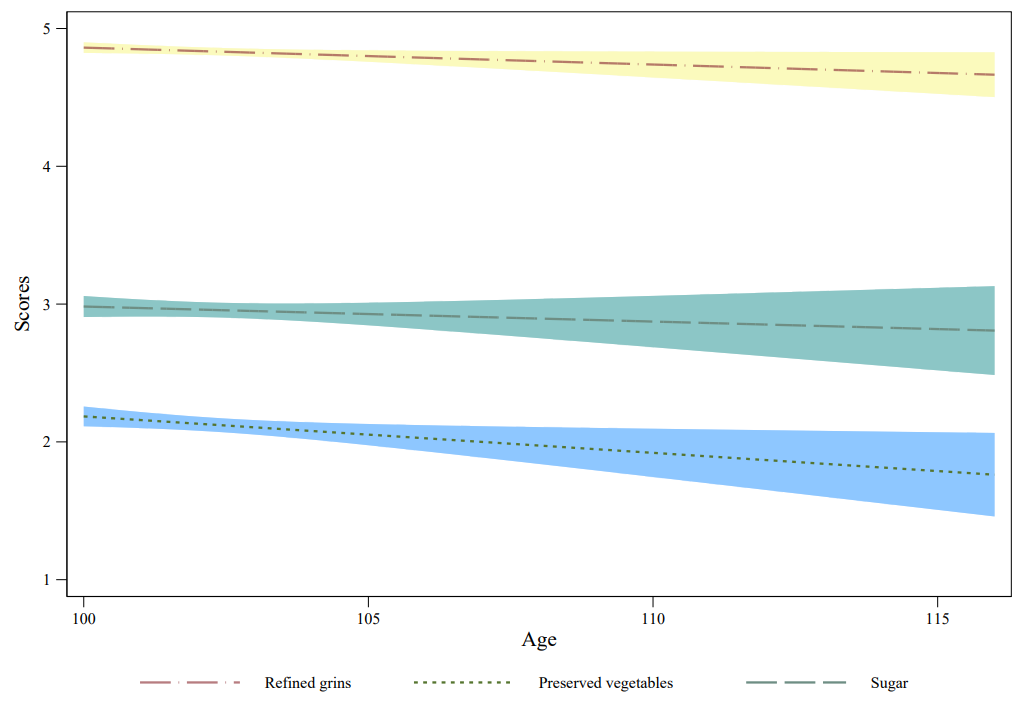


Figure S4 Distribution of Unhealthy Plant Food Scores among Chinese Centenarians in a Cross-Sectional Survey by Age





Figure S5 Distribution of Animal Food Scores among Chinese Centenarians in a Cross-Sectional Survey by Age
